# Adult HIV/AIDS patients are more likely to experience anxiety symptoms

**DOI:** 10.3389/fpsyt.2025.1507020

**Published:** 2025-01-29

**Authors:** Zakir Abdu, Wondwossen Belayneh, Aman Dule, Solomon Seyife Alemu, Lema Fikadu Wedajo, Mohammedamin Hajure, Gebremeskel Mulatu Tesfaye, Yadeta Alemayehu Workneh, Wubishet Gezimu, Mustefa Adem Hussen, Bilisumamulifna Tefera, Sadik Habib

**Affiliations:** ^1^ Department of Psychiatry, Nursing, Midwifery, and Public Health, College of Health Sciences, Mattu University, Mattu, Ethiopia; ^2^ Department of Medicine, Gambella General Hospital, Gambella, Ethiopia; ^3^ Department of Midwifery, and Psychiatry, College of Health Sciences, Madda Walabu University, Sheshemene, Ethiopia; ^4^ Department of Midwifery, Institute of Health Sciences, Wollaga University, Nekemte, Ethiopia; ^5^ Department of Public Health, Mattu Health Sciences College, Mattu, Oromia, Ethiopia

**Keywords:** anxiety, Gambella General Hospital, PLWH, Ethiopia, Mattu University

## Abstract

**Background:**

Among various mental disorders, anxiety disorder is commonly reported in HIV-positive individuals. Compared to the general population, people living with HIV/AIDS exhibit a higher prevalence of anxiety, with an estimated figure of 68.2% *versus* 29% in the general population. However, there is a scarcity of studies on the prevalence and associated factors of anxiety among people living with HIV/AIDS in Ethiopia.

**Method:**

An institution-based cross-sectional study was conducted among 320 participants at Gambella General Hospital. The Beck Anxiety Inventory (BAI) scale, Fagerström Test for Nicotine Dependence (FTND), Severity of Dependence Scale (SDS), and the Alcohol Use Disorder Identification Test (AUDIT) were used to collect the data. Data analysis was performed using SPSS version 25. Bivariate and multivariable logistic regressions were employed to identify independently associated variables, and statistical significance was determined at a *p*-value <0.05.

**Results:**

Out of a total of 323 samples, 320 respondents completed all items, resulting in a response rate of 99.07%. The results showed that 28.4% (95% CI = 23.2–33.9) of participants had anxiety. Factors such as being aged 25 to 40 years, having seen a counselor, HIV disclosure, alcohol use disorders, and perceived high stigma were significantly associated with anxiety in people living with HIV (PLWH).

**Conclusion:**

In the study area, about two out of every seven people living with HIV/AIDS experienced anxiety symptoms. Factors such as being aged 25 to 40 years, having seen a counselor, HIV disclosure, alcohol use disorders, and perceived high stigma were significantly associated with anxiety in this population. Based on these findings, timely intervention is recommended to enhance the overall well-being and quality of life for people living with HIV (PLWH), leading to better health outcomes, reducing the burden of mental health issues, and supporting more holistic, patient-centered care.

## Background

Among various mental disorders, anxiety disorders are one of the most common psychiatric illnesses associated with HIV (1). Anxiety disorders are more than twice as common among HIV+ individuals as in the general population (2). Overall, different studies have reported the incidence of anxiety disorders among people living with HIV/AIDS ranging from 0.6% to 68.2% (3, 4), compared to 29% in the general population (5).

Human immunodeficiency virus (HIV) is a major public health issue that has affected about 39.0 million (33.1–45.7 million) people as of the end of 2022. Of these, an estimated 25.6 million (21.6–30.0 million) people were from Africa (6). An estimated 753,100 people were living with HIV in Ethiopia, with the prevalence being higher among urban populations and women (7).

HIV/AIDS and anxiety disorders have a strong relationship (8). Anxiety disorders affect the prognosis of HIV, delay the time to viral suppression, and increase the rate of antiretroviral failure even after suppression (9). Anxiety may also contribute to behaviors that increase the risk of HIV infection, such as unprotected sex and substance use (10). In addition, anxiety disorders among HIV+ individuals lead to a lower quality of life (11), higher healthcare costs (12), an increased risk of suicide (13), worse health outcomes, and ultimately higher mortality (14). Besides the discomfort caused by anxiety disorders, they can interfere with an HIV+ person’s overall success in managing HIV, as they are a major cause of non-adherence to medication (2).

Although HIV is a public health concern, the mental health of PLHIV is often neglected (6, 15) and still requires more attention and focus. In particular, low-income countries pay almost no attention to or provide no treatment for the mental health of PLWH. This means that the anxiety experienced by PLWH has been largely overlooked.

It is crucial that the current study provides key recommendations to address the many factors associated with anxiety among PLWH and promote the health of the affected population. The aim of this study was to assess the prevalence and underlying causes of anxiety among HIV/AIDS patients at Gambella General Hospital in southwestern Ethiopia.

## Methods

### Study setting

The study was conducted at Gambella General Hospital, which is located in the Gambella Regional State, 760 km southwest of Addis Ababa.

### Study design and period

An institution-based cross-sectional study was conducted from May 10 to 30, 2023.

### Study population

All patients living with HIV who were on follow-up at Gambella General Hospital were included in the sample during the data collection period.

### Eligibility criteria

Inclusion criteria: All adults living with HIV/AIDS, aged 18 years and above, who were on follow-up at the Gambella General Hospital ART clinic were included in the study.

Exclusion criteria: Participants who were unable to respond or were severely ill were excluded from the study.

### Sample size determination

Using a single population proportion formula and the following assumptions—95% confidence level (Zα/2 = 1.96), 5% margin of error (*d* = 0.05), and a prevalence rate of anxiety among PLWHA of 25.6% (16)—the total sample size was calculated. Finally, by adding a 10% non-response rate, the total sample size was 323.

### Sampling technique

A consecutive sampling technique was used to select 323 patients attending the Gambella General Hospital ART clinic during the study period. There were 4,357 adults with 350 ART clients who required follow-up in the ART clinic annually and monthly, respectively. The sample was collected until the required sample size was reached. To avoid repeating patients during the data collection period, if they came for a second visit, their patient card numbers were recorded on the questionnaires and cross-checked.

### Data collection tools

Data was collected using a structured questionnaire by face-to-face interview. The levels of anxiety were assessed using the Beck Anxiety Inventory (BAI) scale. The BAI consists of 21 self-reported items (four-point scale) used to assess the intensity of physical and cognitive anxiety symptoms during the past week. Scores may range from 0 to 63: minimal anxiety levels (0–7), mild anxiety (8–15), moderate anxiety (16–25), and severe anxiety (26–63). Its test–retest reliability (1 week) = 0.75 (17). Perceived stigma was assessed using the short 12-item stigma scale. It was rated on four-point Likert scores (1 = strongly disagree to 4 = disagree), and the score ranged from 12 to 48 (18). In this study, the mean was used as the cutoff point. Participants scoring 22 or above were classified as “high perceived stigma,” whereas scores below 22 were considered “low perceived stigma.”

The Fagerstrom Test for Nicotine Dependence (FTND) was used to assess tobacco use disorder. It has six items, with a total score ranging from 0 to 10 to measure nicotine dependence. A cutoff point ≥5 was considered to indicate tobacco dependence (19, 20). Khat use disorder was assessed by using the tool Severity of Dependence Scale (SDS). It has five items that are scored on a four-point scale (0–3) (21). The alcohol use disorder identification test (AUDIT) was used to assess alcohol use disorders and is developed by the World Health Organization (WHO). The overall rating for AUDIT is 40. A score of 8 or greater is considered to be a drinking disorder (22, 23).

### Data processing and analysis

Once all necessary data were obtained, the data were checked for completeness. Data were edited, cleaned, coded, and entered into EpiData version 3.1. Data were analyzed using SPSS version 25.0. For the analysis of obtained data, simple descriptive statistics were used. Bivariate analysis was used to see the significance of the association. Variables that show a strong association in bivariate analysis were entered into multivariable logistic regressions to control for confounders, and the significance of association was declared at *p*-value 0.05 with 95% confidence interval.

The Hosmer–Lemeshow test *p*-value = 0.126. The logistic regression model fits the data well, as the *p*-value is greater than 0.05. Multicollinearity was checked using variance inflation factors (VIF). The VIF values were less than 2.

## Results

### Socio-demographic and clinical-related factors and substance use characteristics of respondents

From the total of 323 participants, 320 completed all items. Among 320 participants, the majority were women. More than half were Protestant religion followers (**Table 1**). About five out of nine respondents have comorbid medical illnesses such as diabetes mellitus, asthma, TB, and hypertension (**Table 2**). About two-fifths of the participants have a lifetime history of khat chewing, and for every nine respondents, seven of them drink alcohol in their life (**Table 3**).

**Table 1:** Background characteristics of study participants among persons living with HIV/AIDS at Gambella General Hospital, South West Ethiopia, May 2023 (*N =*320).

Study variables	Response	Frequency (*N*)	Percentage (%)
Gender	Male Female	121 199	37.8 62.2
Age (in years)	18–24 25–30 31–40 41 and above	70 84 84 82	21.9 26.3 26.3 25.6
Educational status	Unable to read write Primary education Secondary education College and above	62 125 112 21	19.4 39.1 35.0 6.6
Religion	Protestant Muslim Orthodox Catholic	170 21 99 30	53.1 6.6 30.9 9.4
Ethnicity	Nuer Agnwak Amhara Oromo Others^a^	158 113 24 14 11	49.4 35.3 7.5 4.4 3.4
Marital status	Single Married Divorced Widowed	128 101 73 18	40 31.6 22.8 5.6
Job	Private Farmer Employed Daily labor Driver No job	146 42 83 7 17 28	45.6 13.1 25.9 2.2 4.4 8.8
Residence	Rural Urban	75 245	23.3 76.6
Living arrangement	Alone With family	63 257	19.7 80.3
Monthly income	Less than 1,500 ETB 1,500 ETB and above	47 243	14.7 85.3

a Tigre, Majang, and Gurage.

**Table 2:** HIV related clinical characteristics of study participants among persons living with HIV, Gambella General Hospital, South West Ethiopia, May 2023 (*N =*320).

Study variables	Category/response	Frequency (*N*)	Percentage (%)
Seen by counselor	Yes No	54 266	16.9 83.1
HIV status of partner	Unknown Positive Negative	54 183 83	16.9 57.2 25.9
HIV status disclosure to others	Unknown Not disclosed Disclosed	93 100 127	29.1 31.3 39.7
Comorbid illness^a^	Yes No	173 147	54.1 45.9
CD4 count	Less than 500 500 and above	110 210	34.4 65.6
Perceived stigma	High perceived stigma Low perceived stigma	177 143	55.3 44.7

a Diabetes mellitus, hypertension, asthma, and TB.

**Table 3:** Substance use characteristics of study participants among persons living with HIV, Gambella General Hospital, South West Ethiopia, May 2023 (*N =*320).

Study variables	Category/response	Frequency (*N*)	Percentage (%)
Life time cigarette smoke	Yes No	213 107	66.6 33.4
Cigarette use disorder	Yes No	43 277	13.4 86.6
Life time khat chewing	Yes No	128 192	40.0 60.0
Khat use disorder	Yes No	81 239	25.3 74.7
Life time alcohol use	Yes No	252 68	78.8 21.3
Alcohol use disorder	Yes No	136 184	42.5 57.5

### Prevalence of anxiety among PLWH

The prevalence of anxiety among adult people living with HIV/AIDS at Gambella General Hospital using the Beck Anxiety Inventory scale was 28.4.

### Factors associated with anxiety among PLWH

Marital status, educational level, living arrangement, income per month, duration of being diagnosed with HIV/AIDS, cigarette use disorder, khat use disorder, and having another comorbid medical disorder were not significantly associated with having anxiety in the bivariate logistic regression. However, sex, residence, HIV status of partner, and number of CD4 count showed an association only in the bivariate analysis but did not show a significant association in the final model.

Multivariable logistic regression analysis revealed that age, seen by a counselor, HIV disclosure, alcohol use disorder, and perceived high stigma had a significant association with anxiety in PLWH (**Table 4**). Moreover, chi-square tests were conducted (**Table 5**). Regarding the interaction term tests (*B* = 0.104, *p* = 0.848), the effect of sex on anxiety did not change based on alcohol use disorder (**Table 6**).

**Table 4:** Factors associated with anxiety by bivariate and multivariate logistic regression among PLWH, Gambella General Hospital, South West Ethiopia, May 2023 (*N =*320).

Study variables	Anxiety		COR (95% CI)	AOR (95% CI)	*P*-value
	Yes 91 (28.4%)	No 229 (71.6%)			
Sex					
Female Male	65 (32.7) 26 (21.5)	134 (67.3) 95 (78.5)	1.77 (1.05, 2.99)* Ref	1.81 (0.93, 3.53) Ref	0.07
Age (in years)					
18–24 25–30 31–40 41 and above	14 (20.0) 28(33.3) 35 (41.7) 14 (17.1)	56 (80.0) 56 (66.7) 49 (58.3) 68 (82.3)	1.21 (0.53, 2.76)# 2.29 (1.16, 5.05)* 3.46 (1.68, 7.13)* Ref	1.28 (0.47, 3.49) 2.56 (1.10, 5.94) 4.52 (1.89, 10.82) Ref	0.62 **0.028** **0.001**
Residence					
Rural Urban	29 (37.7) 62 (25.5)	48 (62.3) 181 (74.5)	1.76 (1.02, 3.04)* Ref	1.35 (0.68, 2.68) Ref	0.37
Seen by counselor					
Yes No	84 (31.6) 7 (13.0)	182 (68.4) 47 (87.0)	Ref 3.09 (1.34, 7.14)*	Ref 3.09 (1.20, 7.96)	**0.019**
HIV status of partner					
Not known Positive Negative	32 (59.3) 38 (20.8) 21 (25.3)	22 (40.7) 145 (79.2) 62 (74.7)	4.29 (2.06, 8.95)** 0.77 (0.42, 1.42)* Ref	0.82 (0.25, 2.66) 0.22 (0.08, 1.60) Ref	0.75 0.03
HIV disclosed to others					
Not known Not disclosed Disclosed	38 (40.9) 36 (36.0) 17 (13.4)	55 (59.1) 64 (64.0) 110 (86.6)	4.47 (2.31, 8.62)** 3.64 (1.89, 6.99)** Ref	4.96 (1.74, 14.12) 7.57 (2.73, 20.96) Ref	**0.003** **<0.001**
Alcohol use disorder					
No Yes	40 (21.7) 51 (37.5)	144 (78.3) 85 (62.5)	Ref 2.16 (1.32, 3.53)*	Ref 2.01 (1.11, 3.64)	**0.021**
CD4 count					
Below 500 Above 500	40 (36.4) 51 (24.3)	70 (63.6) 159 (75.7)	1.78 (1.08, 2.93)* Ref	1.68 (0.91, 3.09) Ref	0.09
Perceived stigma					
Low perceived stigma High perceived stigma	25 (17.5) 66 (37.3)	118 (82.5) 111 (62.7)	Ref 2.80 (1.65, 4.76)**	Ref 2.50 (1.35, 4.64)	**0.004**

*p < 0.05; **p < 0.001; #*p* > 0.05 (in bivariate model).

a p-value which is less than 0.05. Bold values indicate p < 0.05.

**Table 5:** Chi-square tests among PLWH, Gambella General Hospital, South West Ethiopia, May 2023 (*N =*320).

Study variables	Anxiety		df	*P*-value (chi-square tests)
	Yes 91 (28.4%)	No 229 (71.6%)		
Sex				
Female Male	65 (32.7) 26 (21.5)	134 (67.3) 95 (78.5)	1	0.032
Residence				
Rural Urban	29 (37.7) 62 (25.5)	48 (62.3) 181 (74.5)	1	0.039
Seen by counselor				
Yes No	84 (31.6) 7 (13.0)	182 (68.4) 47 (87.0)	1	0.006
Alcohol use disorder				
No Yes	40 (21.7) 51 (37.5)	144 (78.3) 85 (62.5)	1	0.002
CD4 count				
Below 500 Above 500	40 (36.4) 51 (24.3)	70 (63.6) 159 (75.7)	1	0.023
Perceived stigma				
Low perceived stigma High perceived stigma	25 (17.5) 66 (37.3)	118 (82.5) 111 (62.7)	1	**<**0.001

**Table 6:** Interaction term analysis among PLWH, Gambella General Hospital, South West Ethiopia, May 2023 (*N =*320).

Variables	*B*	SE	Wald	df	*P*-value
Sex (male vs. female) Alcohol (yes vs. no) Sex * alcohol use interaction	0.511 0.693 0.104	0.384 0.447 0.543	1.768 2.402 0.037	1 1 1	0.184 1.121 0.848
Perceived stigma (low vs. high) Residence (rural vs. urban) Perceived stigma * residence interaction	0.993 0.474 0.068	0.317 0.502 0.610	9.824 0.89 0.013	1 1 1	0.002 0.346 0.911
Seen by counselor (yes vs. no) Alcohol (yes vs. no) Seen by counselor * alcohol use interaction	0.357 -1.315 2.241	0.489 1.121 1.153	0.533 1.376 3.776	1 1 1	0.466 0.241 0.052
Sex (male vs. female) Perceived stigma (low vs. high) Sex * perceived stigma interaction	0.270 0.706 0.512	0.469 0.472 0.577	0.332 2.235 0.788	1 1 1	0.565 0.135 0.375
Perceived stigma (low vs. high) Alcohol use (yes vs. no) Perceived stigma * alcohol use interaction	0.606 1.464 -0.929	0.448 0.484 0.578	12.837 9.138 2.587	1 1 1	<0.001 0.003 0.108
Seen by counselor (yes vs. no) Perceived stigma (low vs. high) Seen by counselor * perceived stigma	1.129 1.044 -0.025	0.771 0.886 0.931	2.144 1.388 0.001	1 1 1	0.143 0.239 0.979

## Discussion

The results from this study indicated that the prevalence of anxiety among the adult population of HIV/AIDS-positive individuals was 28.4%. This finding is consistent with a previous study (16). A possible reason for this could be that both the current and the previous studies were conducted on populations with similar geographical and socioeconomic characteristics. This finding is lower when compared to the previous studies (24, 25). The possible reason might be the difference in population type. The previous study (24) was conducted among clients of voluntary counseling and HIV testing. People may feel very anxious and nervous when they voluntarily undergo a blood test and the virus is detected in their blood. However, our current study was conducted on individuals who were already diagnosed with HIV/AIDS and were undergoing treatment. The difference in study design may also impact the difference in results. The previous study (25) used a case–control design, while the current study employed a cross-sectional design. Another possible reason could be the inclusion of individuals who had not yet started HIV/AIDS treatment in the previous study. Additionally, the previous study was conducted only on individuals over 30 years of age. As is well known, people over 30 often have many responsibilities and experience higher levels of stress. The current finding is higher when compared to the previous study (26, 27). The reason might be that the previous study (27) used a systematic review approach. Additionally, the previous study was conducted in developed countries, where adequate medical services are available and people’s awareness is higher. Another discrepancy in the results is that a longer period of time has passed since the previous study (26) was conducted. As is well known, there is a long-term difference between the two studies, and their results differ. Therefore, these factors may explain why our research results are higher than those of the previous study.

This study showed that participants aged 25 to 40 years were more likely to report an anxiety disorder compared to those aged 41 years and older. This finding is inconsistent with the previous result (28). The possible reason could be that the tool used to assess anxiety and the methods of analysis in the current study differ from those in the previous study. People in their 20s and 30s are adults, and this age group also represents the productive workforce. Additionally, most people get married around this age, and they have responsibilities both to their nation and to their family. They are self-sufficient at this age and often go above and beyond to help others. The study’s findings indicate that patients with HIV/AIDS in this age range who also meet the aforementioned criteria are more likely to develop anxiety than those over 41 years old who also report having anxiety symptoms.

Compared to their peers, the participants who saw the counselor exhibited fewer anxiety symptoms. This finding is supported by the previous study (29). As is well known, counseling helps patients with issues that medicine cannot address. The goal of the counseling process is to help clients become more aware of their mental processes and to teach them how to use useful, adaptable thoughts and behaviors to cope with a variety of situations (30). Patients gain insight into how their thoughts affect their anxiety symptoms during the cognitive portion of therapy. By learning to alter these mental habits, they can reduce the likelihood and severity of anxiety symptoms.

According to the current findings, participants who disclosed their HIV/AIDS status to a third party reported feeling less anxious than those who did not. This finding aligns with a previous study conducted in Zimbabwe, which found that positive disclosure correlates significantly with psychosocial measures (31). HIV disclosure or telling others that you are HIV positive offers many potential benefits. By encouraging partner HIV testing and increasing condom negotiation and use, HIV partner disclosure can reduce the amount of unprotected sexual activity. It can also encourage individuals to disclose their status to partners or others (32, 33). If people stop hiding their illnesses and reach out to those in need, they will eventually receive care.

In this study, compared to individuals without alcohol use disorders, those with alcohol use disorders experienced higher levels of anxiety. This finding is also supported by a previous study, which stated that individuals who used substances had higher anxiety levels (34). The symptoms of anxiety and alcohol use often coincide and overlap (35). Improper use of antiretroviral medication persists even after alcohol consumption. Other mental disorders, such as anxiety, may arise as a direct or indirect result of improper medication use (36). Therefore, alcohol users tend to experience a wider range of anxiety symptoms.

Still this study found that individuals with HIV/AIDS who perceived stigma as strong reported more anxiety symptoms than those who did not. This result is consistent with a previous study (37). HIV/AIDS stigma refers to the negative attitudes, thoughts, and feelings that individuals living with the virus have about themselves as a result of their HIV condition (38). There is a link between mental health issues and the stigma surrounding HIV. When people are concerned about something, they are more likely to experience mental health problems, including anxiety. As a result, the majority of stigmatized HIV/AIDS participants reported experiencing anxiety.

## Conclusion

At Gambella General Hospital, two out of every seven people living with HIV (PLWH) reported anxiety symptoms. Multivariable logistic regression analysis revealed that being aged 25 to 40 years, seeing a counselor, HIV disclosure, alcohol use disorders, and perceived high stigma were significantly associated with anxiety in this population. This study highlights the significant prevalence of anxiety among people living with HIV (PLWH), underscoring the critical need for routine anxiety screening in this population. The findings emphasize the importance of further research to better understand the causal relationships between anxiety and other variables in this context. By integrating mental health services into ART clinics, healthcare providers can offer more comprehensive care, addressing both physical and mental health needs. Routine anxiety screening would not only improve early detection but also ensure timely intervention, ultimately enhancing the overall well-being and quality of life for PLWH. This approach could lead to better health outcomes, reduce the burden of mental health issues, and support more holistic, patient-centered care.

### Limitation of the study

A limitation of this study is that the sample size was small and drawn from a single general hospital, which may not be representative of the broader population. As a result, the findings may have limited generalizability to other hospitals or healthcare settings, especially those in different geographical areas or with differing patient demographics. Therefore, the conclusions drawn from this study may not fully apply to populations outside the specific context of the study’s location or to the general population.

## Data Availability

The raw data supporting the conclusions of this article will be made available by the authors, without undue reservation.
